# Ginsenoside Rh4 Suppresses Metastasis of Esophageal Cancer and Expression of c-Myc via Targeting the Wnt/β-Catenin Signaling Pathway

**DOI:** 10.3390/nu14153042

**Published:** 2022-07-25

**Authors:** Jun Chen, Zhiguang Duan, Yannan Liu, Rongzhan Fu, Chenhui Zhu

**Affiliations:** 1 Shaanxi Key Laboratory of Degradable Biomedical Materials, School of Chemical Engineering, Northwest University, 229 North Taibai Road, Xi’an 710069, China; cehnjun151@163.com (J.C.); duanzhiguang@nwu.edu.cn (Z.D.); liuyannan@nwu.edu.cn (Y.L.); furongzhan@nwu.edu.cn (R.F.); 2 Shaanxi R&D Center of Biomaterials and Fermentation Engineering, School of Chemical Engineering, Northwest University, 229 North Taibai Road, Xi’an 710069, China; 3 Biotech & Biomed Research Institute, Northwest University, 229 North Taibai Road, Xi’an 710069, China

**Keywords:** metastasis, ginsenoside Rh4, ESCC, Wnt/β-catenin, c-Myc

## Abstract

The metastasis of esophageal squamous cell carcinoma (ESCC) is a leading cause of death worldwide, however, it has a poor prognosis. Ginsenoside Rh4 is a rare saponin that has been shown to have potential antitumor effectiveness in ESCC. However, the utility of Rh4 in ESCC metastasis and its undiscovered mode of action has not yet been explored. In this study, we found that Rh4 could inhibit ESCC metastasis by regulating the Wnt/β-catenin signaling pathway and the level of c-Myc, which is an important transcription factor in cancer. In in vitro experiments, Rh4 could inhibit the migration and invasion of ESCC cells without affecting cell viability. In in vivo experiments, Rh4 restrained ESCC metastasis to the lymph nodes and lungs via the suppression of epithelial-mesenchymal transition (EMT). The Wnt agonist HLY78 promoted EMT and migration of ESCC cells, whereas treatment of Rh4 can attenuate the promotion effect of HLY78. The siRNA knocking out c-Myc can also significantly reduce the expression of EMT-related marker proteins. This study illustrates a new concept for further research on the mechanism of Rh4 in ESCC.

## 1. Introduction

Esophageal cancer, as one of the most invasive cancers, has the seventh greatest prevalence worldwide with more than 540,000 cases and ranks sixth in the world for mortality rates [1]. Esophageal squamous cell carcinoma (ESCC) is the most common type of esophageal cancer, comprising more than 90% of cases, and it has a higher incidence in Asia [2]. Due to the lack of effective markers in esophageal cancer screening, most patients are already in stages II or III when esophageal cancer is identified [3]. A large number of patients with primary esophageal cancer have been found to have lymph node metastasis at the first diagnosis [4]. Tumor metastasis endangers the life of patients even after effective treatment [5,6]. Therefore, finding molecular markers and therapeutic intervention targets involved in esophageal cancer metastasis is crucial for guiding treatment and improving the survival rate of esophageal cancer patients.

Recently, research has discerned that the Wnt/β-catenin signal transduction pathway is abnormally activated in cancer stem cells in breast cancer, liver cancer, colon cancer, and other tumor tissues [7]. The Wnt/β-catenin signaling pathway is involved in many cellular activities, consisting of apoptosis, differentiation, senescence, invasion, migration, and epithelial to mesenchymal transition (EMT) [8,9,10]. When the Wnt signal is activated, the increased content of β-catenin enters the cell nucleus to deactivate other proteins, such as vimentin, metal matrix protein MMP-2, etc. [11]. Increasing evidence shows that the Wnt/β-catenin signaling pathway is the main oncogenic pathway of ESCC [12]. Wnt signaling induces EZH2 binding to β-catenin, regulating of the epitranscriptome, including c-Myc [13]. c-Myc is a gene that is highly correlated with cancer, it is involved in tumor initiation, growth, and progression. Moreover, c-Myc contributes to angiogenesis, invasion, and migration [14,15].

EMT is a crucial factor affecting the development and progression of ESCC [16]. Its main characteristic is its ability to decrease the utterances of cell adhesion molecules (such as E-cadherin) and there is also a conversion in the cytoskeleton, closely linked to the expression of vimentin, and the morphological characteristics of mesenchymal cells [17]. Through EMT, converted epithelial cells acquire higher motility, along with MMP2 and MMP9, which are involved in degrading the extracellular matrix in this process [18].

Ginseng is a rare perennial herb and is cyclopedically used in traditional Chinese medicine. Ginsenosides play a crucial part in ginseng, which have antitumor [19], anti-inflammatory [20,21], anti-obesity [22], antidiabetic [23], neuroprotection [24] and immune enhancement effects [25]. Ginsenoside Rh4 is a kind of tetracyclic triterpene saponins and it is composed of triterpene aglycones and glycosides [26]. Previous studies have shown that ginsenoside Rh4 inhibits glycolysis by targeting AKT, which is involved in the suppression of the cell cycle, thereby is effectual in restraining the proliferation of esophageal cancer [27]. We have also previously reported that ginsenoside CK can curb the metastasis of liver cancer [28]. However, whether Rh4 is inhibitory to the metastasis of ESCC remains unknown.

In the study, we evaluated the ability of ginsenoside Rh4 at non-cytotoxic concentrations to inhibit migration, invasion, and EMT of ESCC in vitro and in vivo. Our findings suggested that Rh4 restrains the migration and invasion of ESCC as well as its metastasis to the lymph nodes and lungs through the Wnt/β-catenin pathway-regulated EMT process. Our research shows that Rh4 might be a potential drug for ESCC metastasis measurement.

## 2. Materials and Methods

### 2.1. Cell Culture

The ESCC cell lines KYSE30, KYSE150, and KYSE410 were provided by the Shanghai Institute of Cell Biology. The above cell lines were cultured in a mixed RPMI-1640 medium, containing 10% FBS and 1% penicillin and streptomycin in an incubator at 37 °C with 5% CO_2_.

### 2.2. Antibodies and Reagents

**Figure 1:**
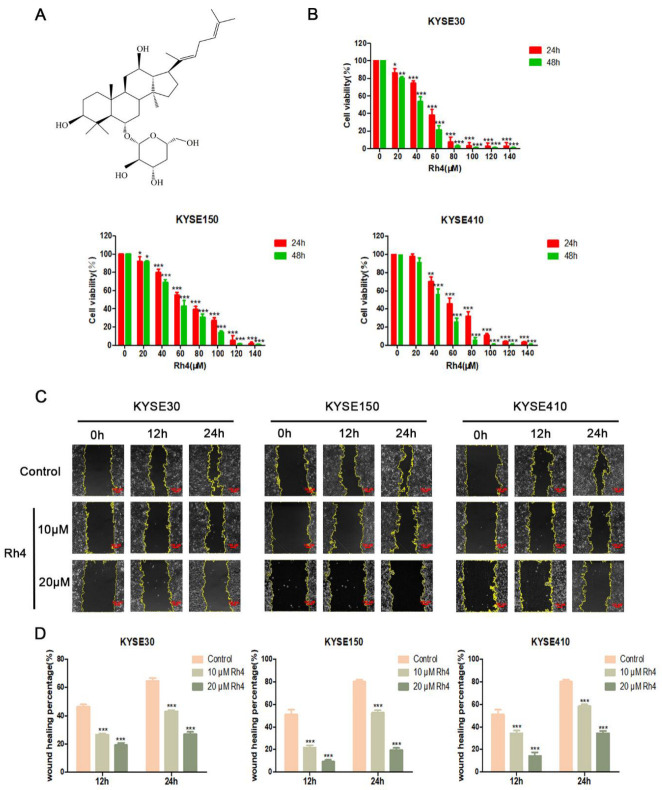
(**A**)The chemical structure of ginsenoside Rh4. (**B**) KYSE30, KYSE150, and KYSE410 were measured with designated content (0–140 µM) of Rh4 cell viability (**C**) Migration images of Rh4 cells with or without the addition of Rh4 cells at 0, 12, and 24 h. The yellow line is the migration edge. Scale bar = 100 µm (**D**) Percent wound healing, treatment group vs. control group. All data are presented as mean ± SD, * *p* < 0.05, ** *p* < 0.01, and *** *p* < 0.001.

Ginsenoside Rh4 (Figure 1A) (purity ≥ 99%) was provided by the Northwestern University Institute of Biomedical Sciences (Xi’an, China). Dimethyl sulfoxide (DMSO) was obtained from Aladdin Biotechnology (Shanghai, China) and methylthiazolyldiphenyl tetra-zolium bromide (MTT) and BCA protein assay reagent kits and phosphatase inhibitor cocktails were obtained from Solarbio Science & Technology Co., Ltd. (Beijing, China). Trypsin, penicillin and streptomycin, crystal violet, and protease inhibitor cocktails were also purchased from Solarbio. Rabbit antibodies against E-cadherin, snail, MMP2, MMP9, Wnt, β-catenin, and c-Myc and mouse antibodies against N-cadherin and Vimentin were obtained from Proteintech Group, Inc (Chicago, MI, USA). Anti-phospho-β-catenin rabbit antibodies, primary antibodies against β-actin, goat anti-mouse IgG, and goat anti-rabbit IgG were purchased from Cell Signaling Technology (Boston, MA, USA). The ratio of related antibodies has been shown (Table S1).

### 2.3. Cell Viability Assays

Cell proliferation was quantified by MTT assays. KYSE30, KYSE150, and KYSE410 cells were seeded onto 1 × 10^4^ cells per well in a 96-well plate. After 24 h, designated destines of Rh4 (0–140 μM) were also placed inside. After the cells were attached, they were measured with Rh4 (0–140 µM) for 24 or 48 h; then, we incubated them with a 50 µL MTT (5 mg/mL) solution for 2–4 h. MTT was then aspirated and replaced with 150 μL of DMSO. The microplate reader (Power Wave XS2, Bio-Tek Instruments Inc., Burlington, VE, USA) was used to measure absorbance at 490 nm.

### 2.4. In Vitro Scratch Assay

We took the KYSE30, KYSE150, and KYSE410 cells in the logarithmic growth phase and inoculated them on a 6-well plate with 1 × 10^6^ cells per well. They were cultivated in an incubator for 24 h. The liter pipette was scratched in the well plate in a three-horizontal and two-vertical manner and the suspended cells were washed with PBS and taken under a microscope for recording. Then, processed cells were measured with ginsenoside Rh4 serum-free medium at a designated content of 0, 10, and 20 μM for 24 h; we took pictures in the same position, with at least four pictures of each area. Cell migration efficiency was assessed by the wound healing rate.

The wound healing rate was calculated by Image J.

### 2.5. Transwell Assays

Migration and invasion assays were assayed using 24-well plates (Corning Incorporated, Corning, NY, USA). In migration experiments, KYSE30, KYSE150, and KYSE410 cells (2 × 105 cells per well) were seeded in the upper compartment of the Transwell chamber with a serum-free conditioned medium (Millipore Corporation, Billerica, MA, USA). A total of 20% FBS medium (600 μL) was added to the lower chamber. Cells were cultured in the presence or absence of Rh4 (10 μM and 20 μM) for 24 h. After incubation, the methanol-fixed filters were stained with crystal violet (0.1%). Then, we took pictures and counted in three randomly selected areas.

The method of the cell invasion assay differs from that of the cell migration assay in that the upper chamber needs to be precoated with 50 μL of Matrigel (Corning, NY, USA).

### 2.6. Quantitative Real-Time PCR (qRT-PCR)

Rh4-treated cells were lysed using TRIzol reagent. They were reverse transcribed with QuantScript RT kit (Tiangen, Beijing, China) and then reverse transcribed using PowerUp SYBR Green Master Mix (Applied Biosystems) on an ABI StepOnePlus Real-Time PCR System (Thermo Fisher Scientific, qRT-PCR was performed in the USA). The 2-^ΔΔ^Ct method was used to evaluate the relative expression.

The primer sequences used were as follows:

E-cadherin (5′-GACGCCATCAACACCGAGTT-3′; 5′-AAATTGCCAGGCTCAATGAC-3′), N-cadherin (5′-GGTGGAGGAGAAGAAGACCAG-3′; 5′-GGCATCAGGCTCCACAGT-3′), Vimentin (5′-GACGCCATCAACACCGAGTT-3′; 5′-CTTTGTCGTTGGTTAGCTGGT-3′), snail (5′-CTTCCAGCAGCCCTACGAC-3′; 5′-CGGTGGGGTTGAGGATCT-3′), Wnt (5′-CAGAGCCACGAGTTTGGAGTT-3′; 5′-GATTGGGTTTGGGTTGGAGGT-3′), β-catenin (5′-GGGATTGGCTTTAGGCCTGT-3′; 5′-GAAATTGCCGTAGCGGGTTC-3′), c-Myc (5′-TGCATGATCAAATGCAACCT-3′; 5′-TCTTTTATGCCCAAAGTCCAA-3′) β-actin (5′-TTGTTACAGGAAGTCCCTTGCC-3′; 5′-ATGCTATCACCTCCCCTGTGTG-3′).

### 2.7. Western Blotting

After washing the cells with PBS buffer, we added RIPA buffer to lyse the cell samples and lymph nodes on ice for 20 min. The supernatant protein concentration was then determined using the BCA protein assay kit (Thermo Scientific, Fremont, CA, USA) by centrifugation at 4 °C and 12,000 rpm for 20 min. Proteins were transferred to polyvinylidene fluoride (PVDF) membranes using SDS-PAGE. Membranes were blocked in TBST containing 5% nonfat milk for 2 h and incubated overnight after adding the primary antibody. Then, visualization was performed using the ECL system (PerkinElmer, Waltham, MA, USA).

### 2.8. Plasmids and siRNA Transfection

According to the human c-myc gene sequence searched in the GenBank database, the corresponding c-myc-siRNA sense: (5′-AGGAAGUCAUCGUGGCCAATT-3′ and antisense: 5′-UUUCUCACUCUCAUACACCTT-3′) primer sequences were devised and commissioned by GenePharma (Shanghai, China). The KYSE30, KYSE150, and KYSE410 cells were diluted in the logarithmic growth phase to 5 × 10^4^/mL, and they were cultured in an antibiotic-free medium. After 48 h of transfection, the cells were exposed to Rh4 in further experiments.

### 2.9. Animal Experiments

Our experiments obtained Northwestern University Animal Ethics Committee approval (NWU-AWC-20210301M). Male BALB/c nude mice (about 20 days, *n* = 8) were obtained from Gem Pharmatech (Jiangsu, China) and fostered under specific pathogen-free (SPF) conditions. After one week of acclimation, the mice were inoculated with 1 × 10^6^ KYSE30 cells to build a footpad transfer model [29]. After the inoculation, the nude mice were left for about 5 days, and then they were placed into different groups: (a) control group (saline, intraperitoneally, i.p.); (b) low-dose Rh4 group (30 mg/kg/day, i.p.); (c) high-dose Rh4 group (60 mg/kg/day, i.p.); (d) capecitabine group (200 mg/kg/day, gavage). In addition, unvaccinated nude mice were differentiated into two groups: (e) normal group (saline, i.p.); (f) normal + Rh4 group (40 mg/kg/day, i.p.). Changes in the body weight of mice were recorded and the body weight was measured every 3 days. After our experiment, the nude mice were sacrificed, and the lymph nodes in the popliteal fossa and main organs were collected for H&E staining.

### 2.10. Hemogram Assay and Measurement of Biochemical Parameters

The peripheral blood we used was obtained from the periocular vein of nude mice and gathered in EDTA-containing specific containers. After blood collection, hematological parameters in each sample, including white blood cell (WBC) count, lymphocyte (LYM) count, and granulocyte (GRAN) count, were measured using an automated hematology analyzer (HC2200, Merrill Lynch, Suzhou, China).

Commercial kits (Shanghai Enzyme Biotechnology Co., Ltd., Shanghai, China) were used to analyze parameters including ALT and AST (liver function) and urea, uric acid, and creatinine (renal function).

### 2.11. Immunofluorescence Assay (IF)

Antigen retrieval in paraffin sections of lymph nodes and lung tissues was performed after specific processing (deparaffinization and dehydration). Fluorescent antibodies were incubated with the second antibodies for 2 h. Fluorescence intensity analysis was visualized and analyzed using a confocal microscope (Tokyo, Japan).

### 2.12. Histopathology and Immunohistochemistry

The lymph nodes and major organs that we needed were isolated from the mice during the course of the disease to form paraffin-embedded sections that were used for hematoxylin and eosin (H&E) staining. For the immunohistochemistry assay, lungs and lymph nodes were stained with E-cadherin, N-cadherin, Vimentin, Snail, c-Myc, Wnt, β-catenin, and p-β-catenin. The images observed were captured with a light microscope (Nikon, Japan).

### 2.13. Statistical Analysis

Experimental data represent mean ± standard deviation (SD). The experimental data were analyzed with SPSS and GraphPad Prism software using a one-way analysis of variance (ANOVA). A value of *p* < 0.05 was considered statistically significant for all experiments.

## 3. Results

### 3.1. Ginsenoside Rh4 Suppresses the Migration and Invasion of ESCC Cells

To examine the effect of ginsenoside Rh4 on the motility of ESCC without affecting cell viability, we conducted an MTT test in vitro of ginsenoside Rh4 on KYSE30, KYSE150, and KYSE410. Three cell lines were measured with unequal contents of Rh4 (0–140 µM) for 24 or 48 h and subjected to MTT. These results demonstrated that the IC50 values of KYSE30, KYSE150, and KYSE410 cells that were treated with Rh4 for 24 h are 52.28 μM, 64.88 μM, and 54.72 μM (Figure 1B). We chose to incubate with ginsenoside Rh4 (10 and 20 μM) to explore its effect on cell motility because the cell viability at those concentrations exceeded 80%.

**Figure 2:**
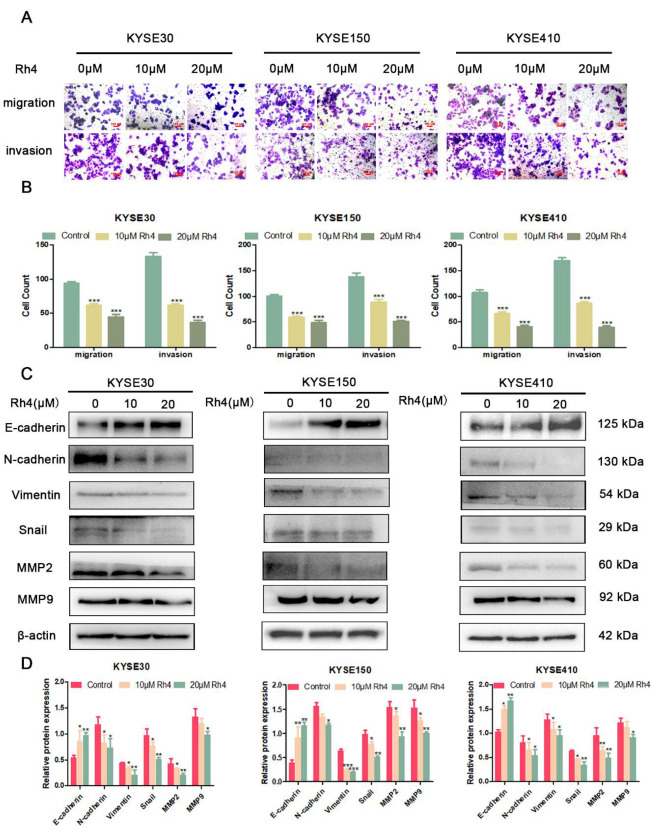
(**A**) Migration and invasion images of KYSE30, KYSE150, and KYSE410 cells. (**B**) Counting cells after crystal violet staining (**C**,**D**) Protein level of E-cadherin, N-cadherin, Vimentin, snail, MMP2, and MMP9 in KYSE30, KYSE150, and KYSE410 after Rh4 treatment (10 and 20 µM). All data are presented as mean ± SD, * *p* < 0.05, ** *p* < 0.01, and *** *p* < 0.001.

The migration and invasion ability of ESCC were assessed using in vitro scratch and Transwell assays. In the in vitro scratch experiment, ginsenoside Rh4 (10 and 20 μM) significantly delayed wound closure (*p* < 0.01) at 12 and 24 h compared to untreated cells in all cell lines tested (Figure 1C). Through in vitro cell movement analysis, we confirmed that ginsenoside Rh4 restrained the migration and invasion of KYSE30, KYSE150, and KYSE410 cells. The quantity of invading cells in the tested cells decreased with increasing Rh4 concentration (Figure 2A). In contrast to the corresponding cells in the control group, the quantity of penetrating cells in the low-dose Rh4 group was reduced by at least 30%, and the quantity of penetrating cells in the high-dose Rh4 group was reduced by more than 50% (Figure 2B). Furthermore, the MMP2 and MMP9 in the cells were also probed. In contrast to the control group, ginsenoside Rh4 can significantly down-regulate the expression of these two proteins (Figure 2C). This demonstrates that ginsenoside Rh4 can potentially inhibit the invasion ability of ESCC.

### 3.2. Ginsenoside Rh4 Inhibits the Metastasis of ESCC by Regulating EMT

**Figure 3:**
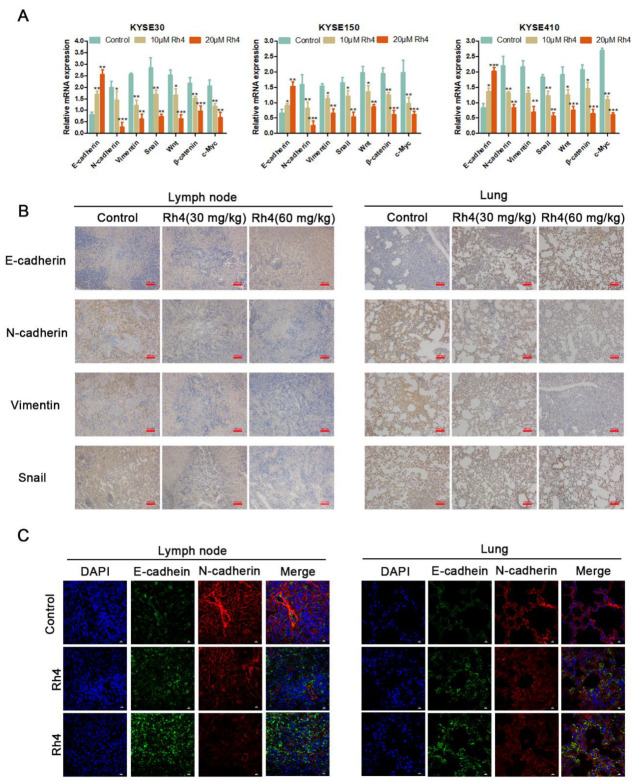
(**A**) Transcript of E-cadherin, N-cadherin, Vimentin, snail, wnt, β-catenin, and c-Myc after Rh4 treatment (10 and 20 µM). (**B**) Immunohistochemical images of E-cadherin, N-cadherin, Vimentin, and snail. In lymph nodes and lungs. Scale bar = 100 µm. (**C**) Immunofluorescence analysis showed that Rh4 promoted E-cadherin in lymph nodes and lungs and inhibited the expression of N-cadherin. Scale bar = 10 µm. All data are presented as mean ± SD, * *p* < 0.05, ** *p* < 0.01, *** and *p* < 0.001.

As previously reported, EMT is involved in the metastasis of multiple cancers [30]. Therefore, to study if Rh4 inhibits the metastasis of ESCC by regulating EMT, we detected the expression of KYSE30, KYSE150, and KYSE410 related proteins including E-cadherin, N-cadherin, vimentin, and snail (with or without Rh4) (Figure 2C). As shown in Figure 2D, treatment with ginsenoside Rh4 decreased the content of N-cadherin, vimentin, and snail, and it improved E-cadherin. To explore the effect of Rh4 on the gene levels of EMT in ESCC, qRT-PCR was used to examine the level of related mRNA in KYSE30, KYSE150, and KYSE410 (with or without Rh4). The results demonstrated that Rh4 also increased mRNA expression. Rh4 had a significant impact reducing N-cadherin, vimentin, and snail, and improving E-cadherin’ expression (Figure 3A). This is consistent with Western blotting experiments.

**Figure 4:**
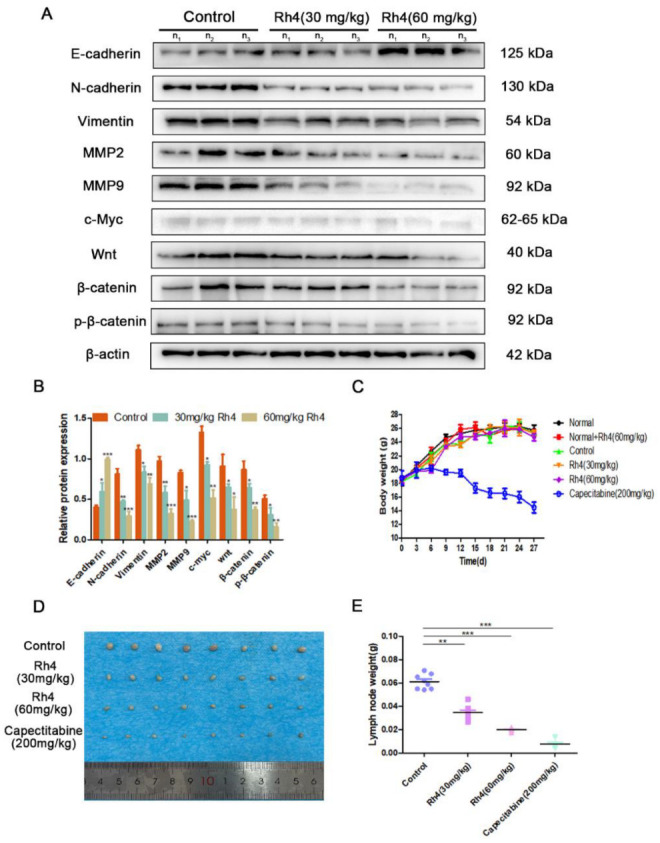
(**A**,**B**) Protein level of E-cadherin, N-cadherin, Vimentin, MMP2, MMP9, Wnt, β-catenin, and c-Myc in the lymph node. (**C**) Body weight change curve of six groups of mice. (**D**) Representative image of KYSE30 popliteal lymph nodes of measured groups. (**E**) Popliteal lymph nodes were measured after sacrifice. All data are presented as mean ± SD, * *p* < 0.05, ** *p* < 0.01, and *** *p* < 0.001.

In order to investigate whether Rh4 also affects ESCC metastasis by curbing EMT in vivo, a mouse footpad lymphatic metastasis model was used to evaluate the role of Rh4. The immunohistochemical results of lymph node and lung tissue illustrated that Rh4 treatment could improve the content of E-cadherin and reduce the positive increase in N-cadherin, Vimentin, and snail (Figure 3B). In the results of immunofluorescence experiments on mouse lymph nodes and lung tissues, the fluorescence intensity of E-cadherin was also significantly heightened, whereas the fluorescence intensity of N-cadherin was significantly lowered, both of these results were in contrast to the control group (Figure 3C). In addition, the expression level of E-cadherin, N-cadherin, Vimentin, and snail in lymph nodes attained the same change trend results in in vitro experiments and were exanimated (Figure 4A,B). The above results demonstrated that Rh4 inhibits the metastasis of ESCC carcinoma by inhibiting EMT.

### 3.3. Ginsenoside Rh4 Inhibits ESCC Metastasis In Vivo and Has Low Toxicity

Next, we studied the effect of ginsenoside Rh4 in the transfer of ESCC in vivo. We injected KYSE30 cells into the left foot pad of male BALB/c mice. While determining lymph-node-associated protein expression, we discerned that MMP2 and MMP9 in the Rh4 measurement group were also notably reduced compared to the control group (Figure 4A). Moreover, no notable diversification in the weight change of mice in the Rh4 treatment group was found in contrast to the normal group, but there was a conspicuous decrease in the capecitabine group (Figure 4C). The lymph nodes were collected and weighed after the experiment was completed. The growth of lymph nodes in the Rh4 group and capecitabine group was restrained. In contrast to the control group, the size and weight of lymph nodes in the 30 mg/kg Rh4 group and 60 mg/kg Rh4 group decreased (Figure 4D,E). Moreover, the number of lung nodules in these two groups was also significantly less than that in the control group (Figure S1). These results indicate that the addition of Rh4 can reduce the metastasis of ESCC cells in the proximal popliteal lymph nodes and distal lung tissue.

**Figure 5:**
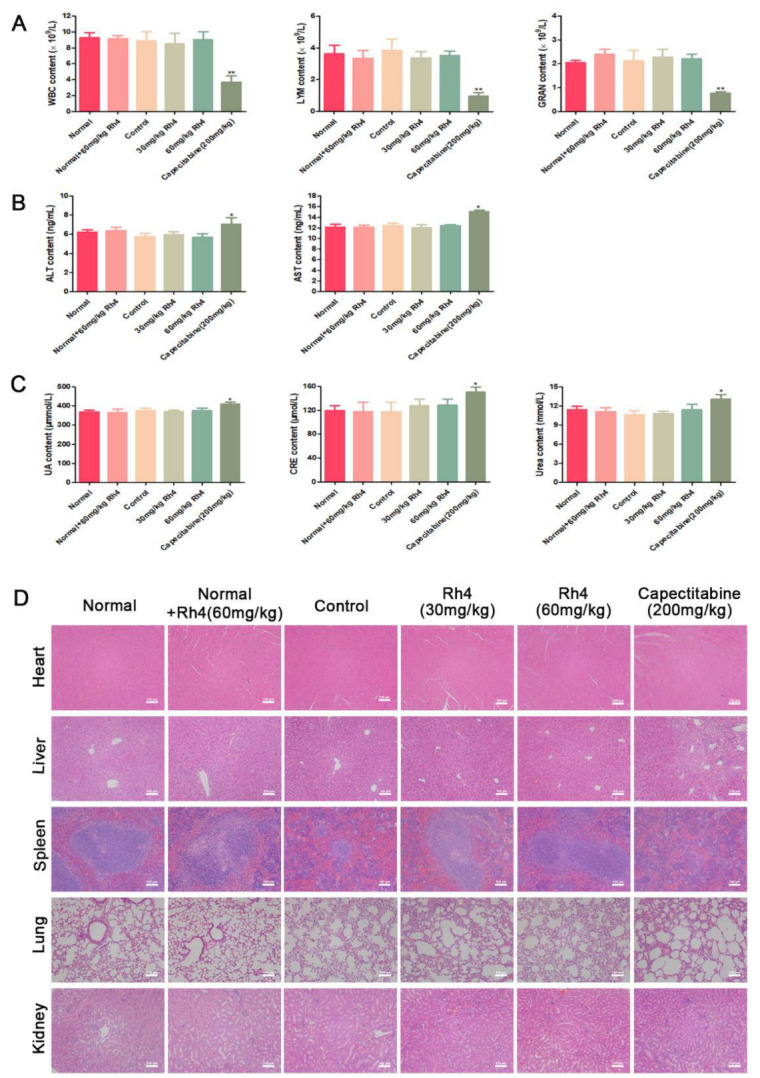
(**A**) The contents of peripheral blood white blood cells (WBC), lymphocytes (LYM), and granulocytes (GRAN) in each group. Liver and kidney function indexes, consisting of (**B**) urea, uric acid, and CRE. (**C**) ALT and AST. (**D**) Images of major organs in each group, consisting of heart, liver, spleen, lung, and kidney. Scale bar = 100 µm. All data are presented as mean ± SD, * *p* < 0.05, and ** *p* < 0.01.

Therefore, to explore the safety of Rh4 in foot pad transfer model mice, we explored the peripheral blood parameters of six groups of mice (WBC, LYM, and GRAN). There were no significant differences in the hematology index of WBC, LYM, and GRAN (Figure 5A). Next, we studied the effect of Rh4 on organ activity. Compared with the normal group, the renal function indexes (urea, uric acid, and creatinine) and liver function indexes (ALT, AST) of mice in the Rh4 treatment group did not change significantly (Figure 5B,C). However, the related indicators in the capecitabine group were conspicuously increased, which suggested that capecitabine has obvious toxicity. H&E staining illustrated that in the normal group, the physiological appearance of important organs in the normal + Rh4 (60 mg/kg) and Rh4 measurement groups showed no obvious pathological changes (Figure 5D). However, in contrast to the control group, liver slices of the capecitabine group showed inflammatory infiltration, and the kidney slices also appeared to be disordered (Figure 5D). Additionally, in the organ index, the spleen index of the capecitabine group also showed an apparent decrease (Table S2). In summary, the results indicated that Rh4 treatment was not significantly toxic in vivo.

### 3.4. Rh4 Inhibits ESCC Metastasis through Wnt/β-Catenin Signaling Pathway

**Figure 6:**
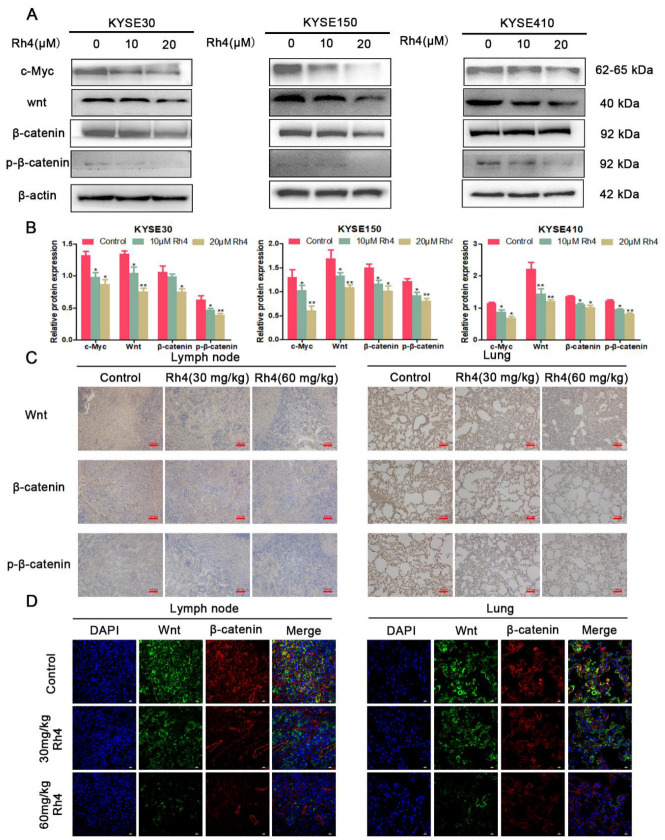
(**A**,**B**) Relative protein levels of Wnt, β-catenin, and p-β-catenin after KYSE30, KYSE150, and KYSE410 were treated by Rh4. (**C**) Immunohistochemical analysis showed that Rh4 attenuated Wnt, β-catenin, and p-β-catenin in lymph nodes and lungs. Scale bar = 100 µm. (**D**) Immunofluorescence analysis showed that Rh4 also attenuated the level of Wnt and β-catenin in lymph nodes and lungs. Scale bar = 10 µm. All data are presented as mean ± SD, * *p* < 0.05, and ** *p* < 0.01.

To investigate if Rh4 inhibits ESCC metastasis through the Wnt/β-catenin pathway, we tested the protein levels of the Wnt/β-catenin pathway. In contrast to the control group, KYSE30, KYSE150, and KYSE410 cells demonstrated different densities of Rh4 reduced Wnt, β-catenin, and p-β-catenin levels (Figure 6A), similar results were also found in the Western blotting experiment of tumor tissues (Figure 6B). In addition, we also found from the immunohistochemical results of lymph node and lung tissue that Rh4 decreased the Wnt/β-catenin interrelated proteins, which was the same as the results of Western blotting experiment (Figure 6C). The immunofluorescence results for lymph node and lung tissue also suggested the Wnt and β-catenin in the Rh4 measurement group were noticeably reduced compared to the control group (Figure 6D).

**Figure 7:**
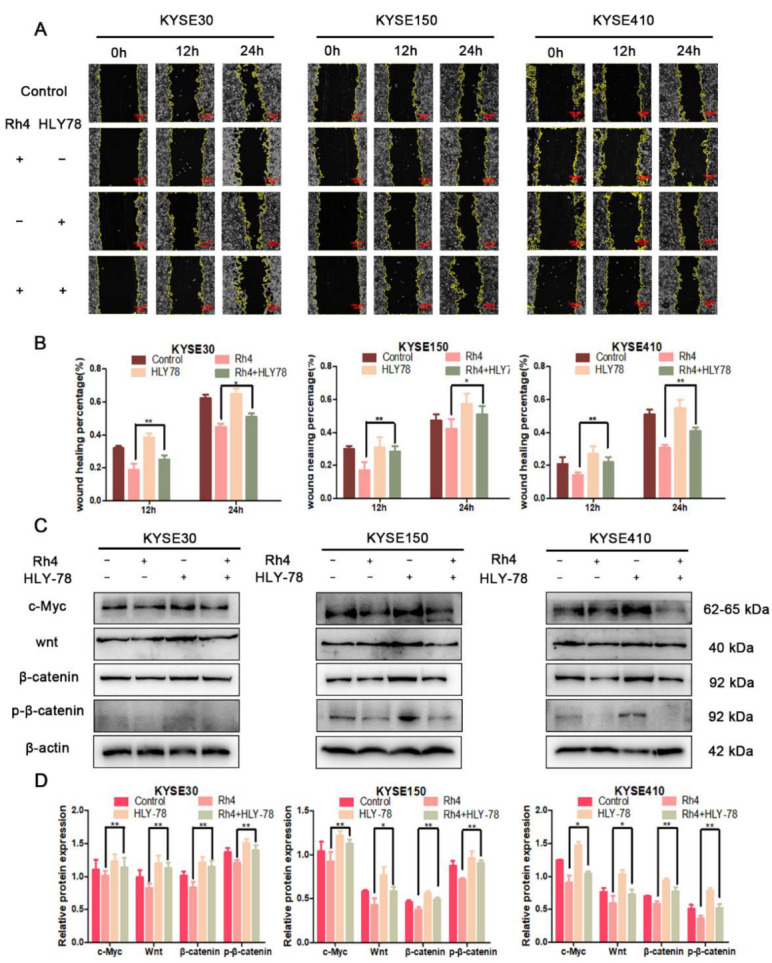
(**A**) Migration images with or without the addition of Rh4(HLY78) at 0, 12, and 24 h. The yellow line is the migration edge. Scale bar = 100 µm (**B**) Percent wound healing, treatment group vs. control group. (**C**) Protein levels after Rh4 and HLY-78 combined treatment of KYSE30, KYSE150, and KYSE410. (**D**) Bar graphs showing protein expression level. All data are presented as mean ± SD, * *p* < 0.05 and ** *p* < 0.01.

In order to further explore the utility of Rh4 in the Wnt/β-catenin signaling pathway, we introduced HLY78, which is an agonist of Wnt. The results we obtained from the scratch test showed that the addition of HLY78 could improve the migration ability of KYSE30, KYSE150, and KYSE410 cells. However, with the combined action of Rh4 and HLY78, the wound healing efficiency of the three types of cells was betwixt the control group and the HLY78 group (Figure 7A,B). In addition, we also examined the results in the Wnt/β-catenin pathway proteins after adding HLY78 (Figure 7C). The results showed that the levels of Wnt, β-catenin, and p-β-catenin interrelated proteins are consistent with the expression trend of the scratch experiment (Figure 7D), which indicated that Rh4 restrained the metastasis of ESCC by inhibiting the Wnt/β-catenin pathway.

### 3.5. c-Myc Is Essential in the Inhibition of ESCC Metastasis by Rh4

**Figure 8:**
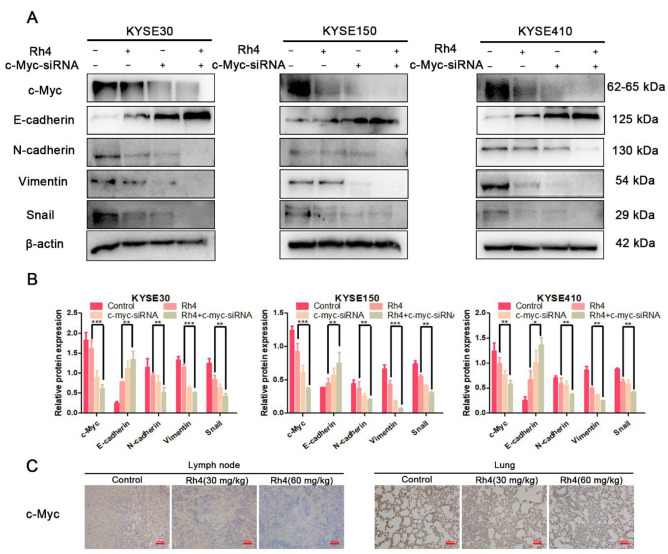
(**A**,**B**) Addition of c-Myc-siRNA enhanced Rh4 inhibition of E-cadherin, N-cadherin, Vimentin, and snail (**C**) Rh4 reduces c-Myc expression in lymph nodes and lungs in vivo. Scale bar = 100 µm. All data are presented as mean ± SD, * *p* < 0.05, ** *p* < 0.01, *** *p* < 0.001.

It has been reported that the expression of c-myc can be adjusted by the Wnt/β-catenin signaling pathway. For our experiments, we confirmed that Rh4 could decrease c-Myc in KYSE30, KYSE150, and KYSE410 (Figure 6A). In order to investigate the utility of Rh4 on c-Myc in ESCC, we used c-Myc-siRNA to silence c-Myc, and then determine the protein level of EMT. These results demonstrated that c-Myc-siRNA significantly decreased c-Myc’s expression. Silencing c-Myc reduced N-cadherin, Vimentin, and snail and improved the E-cadherin. Additionally, the combined effect of Rh4 and c-Myc-siRNA was more obvious than that of c-Myc (Figure 8A,B). The immunohistochemistry of the lymph nodes and lung tissues also illustrated the same results (Figure 8C). The results revealed that c-Myc is an essential transcription factor in the inhibition of ESCC metastasis by Rh4.

## 4. Discussion

Metastasis, which accompanies the diffusion of cancer cells from the initial part of the disease to distant sites [31], includes angiogenesis, cell adhesion, ECM degradation, migration, invasion, and transportation to additional areas [32]. ESCC is powerfully aggressive, and it causes high mortality [33]. A large number of patients with primary esophageal cancer have been found to have lymph node metastasis at the initial diagnosis [34]. Cancer has invaded the muscle layer in most patients, and at least half of all cancers have metastasized to other tissues and organs, most notably the lymph nodes and lungs [35]. It has been shown in recent studies that Ginsenoside Rk1 has an inhibitory effect on the expression of PD-L1 in lung adenocarcinoma [36]. Ginsenoside CK inhibits migration and invasion of human osteosarcoma cells and TGF-β-induced A549 cells via PI3K/mTOR/p70S6K1 and SIRT [37,38]. Our previous research has discerned that Rh4 has a significant effect on the suppression of the proliferation of esophageal cancer. In our research, we verified that Rh4 possesses an inhibitory function on ESCC metastasis. Scratch experiments showed that ginsenoside Rh4 had an obvious inhibitory effect on the migration of ESCC. When 20μM Rh4 was applied for 24 h, the wound healing rate of all cells was reduced by more than 60%. In the KYSE30 transplanted foot pad mouse model, we determined that 30 and 60 mg/kg Rh4 significantly curbed the growth of lymph nodes. Moreover, the ginsenoside group had a reduced expression of metastasis-related proteins in lymph nodes and lung tissues. Based on these results, Rh4 has a significant outcome for ESCC metastasis in vitro and in vivo.

Capecitabine is a first-line drug in the measurement of ESCC metastasis, but its many secondary actions, including liver toxicity and nephrotoxicity, limit the safe dose of capecitabine for patients [39]. If the adverse reaction is too strong, the drug may even be stopped [40]. Natural medicine may offer a new approach to the measurement of esophageal squamous cell carcinoma metastasis. Previous reports have demonstrated that capecitabine could cause liver damage [41,42,43], kidney damage, and even cardiotoxicity. The relevant serum biochemical indicators we obtained revealed organ damage, with liver and kidney activity (CRE, UA, and urea). H&E staining of various organs and tissues after capecitabine treatment also showed liver damage and spleen damage. Moreover, the measurement of blood parameters illustrated that capecitabine significantly reduced the number of immune cells (LYM, WBC, and GRAN), indicating capecitabine caused blood toxicity and affected the immune function of mice. Relevant indicators and H&E staining pictures of Rh4 group mice indicated that Rh4 did not influence the organ function of mice. Additionally, it had no obvious effect on the number of immune cells, which reveals Rh4 has no hematological toxicity. The results of the organ index showed that the spleen and lung indices of the mice were significantly reduced. The limitations of capecitabine on the immune responses and lung function in mice are noteworthy [44]. These indicators did not change significantly in the Rh4 group. Thus, these results suggest that Rh4 has minimal side effects in the treatment of ESCC metastasis in ESCC.

As previously reported, the Wnt/β-catenin signaling pathway is an oncogenic driving pathway in ESCC, which has been widely reported [45]. Wnt is a crucial part of cell axis patterning, including movement. β-catenin (β series protein) is an adhesion factor, and recent studies have found that it has the dual function of regulating and coordinating cell adhesion and gene transcription [46,47]. HLY78 is an agonist of the Wnt/β-catenin signaling pathway. HLY78 can enhance Wnt signaling by targeting the DIX domain of Axin in the channel and enhancing Axin-LRP6 cross-linking [48,49,50]. We pretreated with the Wnt agonist HLY78 to investigate the crucial utility of ginsenoside Rh4. The results of the scratch test discerned that after adding HLY78, the motility of ESCC cells was improved in contrast to the control group. Under the joint action of Rh4, the wound healing rate was between that of the Rh4 group and the HLY78 group. Western blotting illustrated that Wnt, β-catenin and p-β-catenin were consistent with the results of the scratch experiment. Previously, c-Myc expression was shown to be adjusted by the Wnt/β-catenin signaling pathway [51,52]. Moreover, after adding c-Myc-si-RNA, the expression of several EMT-related proteins showed a more obvious trend of change, compared with when only Rh4 was added. This indicates that Rh4 can restrain the metastasis of ESCC by inhibiting the Wnt/β-catenin signaling pathway.

## 5. Conclusions

**Figure 9:**
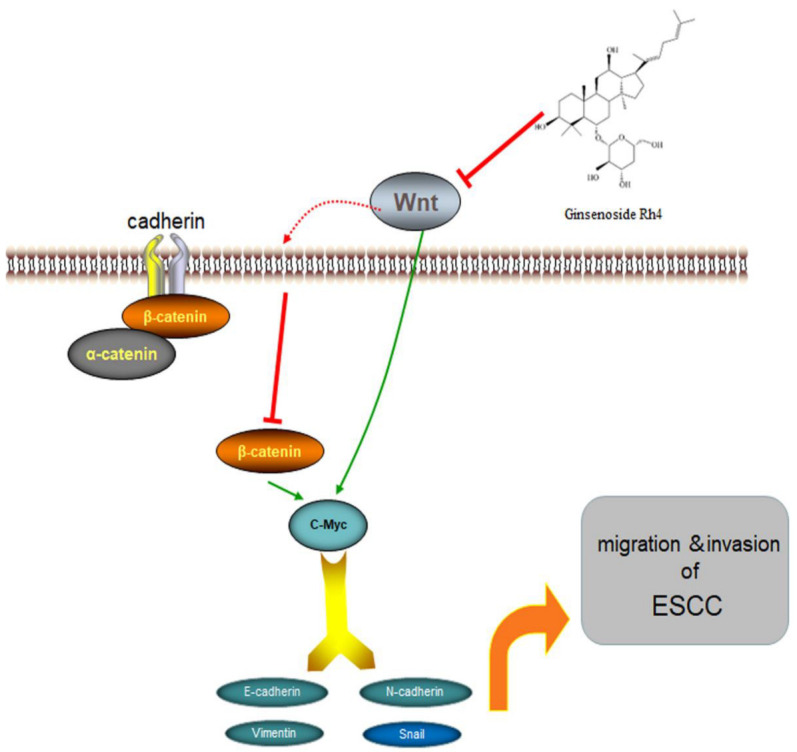
Proposed molecular mechanism of Rh4 anti-metastasis of ESCC.

To summarize, the research reveals for the first time that ginsenoside Rh4 restrains the metastasis of ESCC cells by adjusting the Wnt/β-catenin signaling pathway (Figure 9). In the mouse footpad lymph node metastasis model, Rh4 shows obvious antitumor metastasis activity with few side effects. Our study offers new perspectives on Rh4 as a latent anti-metastatic medicine.

## Data Availability

The original contributions presented in the study are included in the article and supplementary materials. Further inquiries can be directed to the corresponding author(s).

## Supplementary Materials

The following supporting information can be downloaded at: https://www.mdpi.com/article/10.3390/nu14153042/s1. Table S1: Information of antibodies; Table S2 Date of organ index; Figure S1:(A) KYSE30 cells were injected into the footpads of BALB/c mice, and lungs were harvested for staining. (B)Tumor nodule counts in lung tissue.
