# Supplementary material for: Ginsenoside Rh4 Suppresses Metastasis of Esophageal Cancer and Expression of c-Myc via Targeting the Wnt/β-Catenin Signaling Pathway
Source: Nutrients. 2022 Jul 25;14(15):3042. doi: 10.3390/nu14153042 (PMC9331240; doi:10.3390/nu14153042)
Supplement: Supplementary file 1 [file nutrients-14-03042-s001.zip › nutrients-1782780-supplementary.pdf]

Ginsenoside Rh4 suppresses metastasis of esophageal cancer and expression of c-Myc via targeting the Wnt/β-catenin signaling pathway

Jun Chen[a, b, c], Zhiguang Duan[a, b, c], Yannan Liu[a, b, c], Rongzhan Fu[a, b, c], Chenhui Zhu*[a, b, c].

[a]Shaanxi Key Laboratory of Degradable Biomedical Materials, School of Chemical Engineering,Northwest University, 229 North Taibai Road, Xi'an, Shaanxi 710069, China

[b]Shaanxi R&D Center of Biomaterials and Fermentation Engineering, School of Chemical Engineering, Northwest University, 229 North Taibai Road, Xi'an, Shaanxi 710069, China

[c]Biotech. & Biomed. Research Institute, Northwest University, 229 North Taibai Road, Xi'an, Shaanxi 710069, China

*Address correspondence to: Chenhui Zhu, E-mail: zch2005@nwu.edu.cn, Tel./fax: +86-29-88305118.

Table S1 Information of antibodies

Antibody	Immunofluorescence	Immunohistochemistry	Western blot	Manufacturer	Molecular Weight (kDa)	Cat. Number
Wnt	1:500	1:100	1:1000	PTG	60	55184-1-AP-
β-catenin	1:200	1:1000	1:5000	PTG	92	51067-2-AP
p-β-catenin	1:100	1:200	1:1000	CST	92	#4176
c-Myc	1:100	1:500	1:1000	PTG	62	10828-1-AP-
MMP2	---	---	1:1000	PTG	60	10373-2-AP
MMP9	---	---	1:1000	PTG	90	10375-2-AP
N-Cadherin	1:300	1:4000	1:10000	PTG	130	22018-1-AP
E-Cadherin	1:300	1:4000	1:10000	PTG	120	20874-1-AP
Vimentin	---	1:2000	1:1000	PTG	54	10366-1-AP
Snail	---	1:800	1:1000	PTG	29	13099-1-AP
β-actin	---	---	1:2000	PTG	42	20536-1-AP

Table S2 Date of organ index

The date of organ index in the normal group, the normal group + Rh4 group, the control group, the Rh4 low-dose group, the Rh4 high-dose group and the Capecitabine group.

Organ index	Normal	Normal+Rh4(40mg/kg)	Control	Rh4(30mg/kg)	Rh4(60mg/kg)	Capectitabine(200mg/kg)
Heart	0.76±0.07	0.67±0.03	0.75±0.05	0.69±0.03	0.73±0.02	0.81±0.03
Liver	5.62±0.36	6.05±0.38	6.12±0.33	6.31±0.48	6.09±0.37	5.73±0.29
Spleen	1.21±0.06	1.30±0.05	1.44±0.23	1.15±0.25	1.33±0.17	0.52±0.04
Lung	1.06±0.17	1.00±0.18	2.17±0.32	0.79±0.02	0.93±0.09	2.12±0.23
Kidney	1.69±0.03	1.80±0.16	1.68±0.19	1.81±0.08	1.73±0.07	1.79±0.16

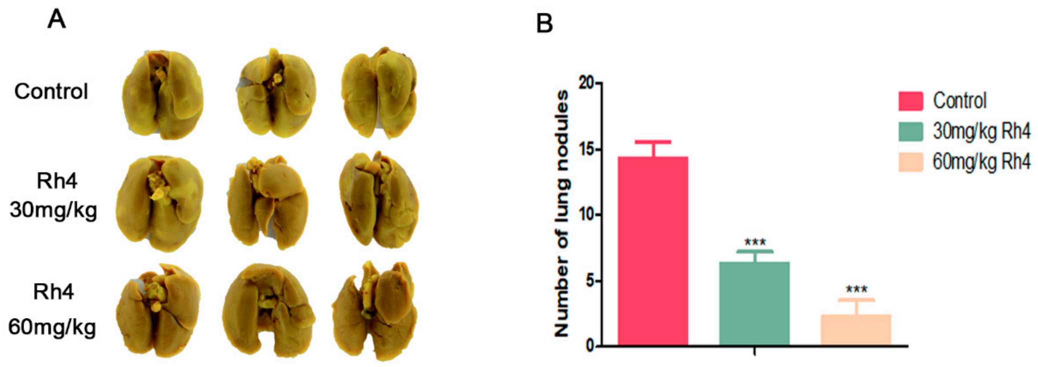

Figure S1. (A) KYSE30 cells were injected into the footpads of BALB/c mice, and lungs were harvested for staining. (B)Tumor nodule counts in lung tissue.
